# Pathways leading to coronary revascularisation among patients with diabetes in Finland: a longitudinal register-based study

**DOI:** 10.1186/1472-6963-11-180

**Published:** 2011-08-03

**Authors:** Tuulikki Vehko, Reijo Sund, Kristiina Manderbacka, Unto Häkkinen, Ilmo Keskimäki

**Affiliations:** 1 Health and Social Services, Service Systems Research Unit, National Institute for Health and Welfare (THL), Helsinki, Finland; 2 Centre for Health and Social Economics CHESS, National Institute for Health and Welfare (THL), Helsinki, Finland

## Abstract

**Background:**

Chronic conditions such as coronary heart disease (CHD) challenge health care to provide systematic and long-lasting disease management. In this study of patients who were revascularized, we examine whether treatment pathways leading to coronary revascularisation differ between patients with and without diabetes.

**Methods:**

This retrospective, nationwide register-based study in Finland in 1998-2007 describes temporal trends in the proportions of 1) revascularisations performed at the first treatment period, and 2) suboptimal treatment pathways to revascularisations, i.e. pathways containing several cardiac emergency hospitalisations. Differences between patient groups were examined using a logistic regression model adjusting for age, comorbidity, and region.

**Results:**

Among patients who underwent revascularisation, upward trends were found in the proportions of revascularisations performed during first hospital admission: among men with CHD alone, the percentages were 28% in 1998 and 77% in 2007; among men with insulin-dependent diabetes (IDD) they were 16% vs. 58% for the respective years; and among men with non-insulin dependent diabetes (NIDD) they were 25% vs. 69%, respectively. Among women the percentages were for non-diabetic group 32% vs. 77%; for IDD group 36% vs. 64%; and for NIDD group 33% vs. 73% for the respective years. Patients with diabetes were less likely to undergo revascularisation during the first hospital admission, in 2005-2007, the odds ratio (OR) for IDD among men was 0.52 (95% confidence interval 0.42-0.64) and for NIDD among men it was 0.79 (95% CI 0.73-0.86) compared to patients with CHD alone. The respective ORs among women were 0.59 (95% CI 0.44-0.78), and 0.83 (95% CI 0.74-0.93).

**Conclusions:**

Treatment practices changed substantially during the study period to favour performing revascularisation during the first hospital admission. The large increase in coronary angioplasty operations is likely to be an important factor behind these changes. However, fewer operations are performed during the first CHD hospitalisation of diabetic patients who undergo coronary revascularisation and they experience more often emergency hospital admissions before the operation than patients without diabetes. To avoid adverse cardiac events, more attention is needed in managing diabetic CHD patients' referral pathways to revascularisation.

## Background

Research evidence suggests that coronary heart disease (CHD) treatment among patients with diabetes is not completely in line with clinical guidelines. Furthermore, treatment has been reported as not being as intensive as among patients without diabetes [1-3]. In Finland medication to prevent adverse CHD events has been reported to be suboptimal for hospitalized diabetic persons with myocardial infarction (MI) and for ambulatory CHD patients [4-6]. Similarly, access to revascularisation among patients with diabetes has been found to be poorer than among patients without diabetes [7].

In Finland a notable increase of resources to perform revascularisations took place in the 1990s and a national diabetes prevention and treatment programme was launched in 2000. The programme prominently underlines diabetes as a vascular disease; patients without any previous coronary events but diagnosed with diabetes are considered to display an increased risk for fatal coronary event equivalent to that among patients with a previous MI [7-9].

However, little is known of the development of treatment practices in coronary care and whether changes in these practices have had an effect on pathways of treatment leading to coronary revascularisation for different patient groups. More information is also needed on whether the increase in resources for invasive cardiac treatment and the implementation of the national diabetes programme have been followed by changes in treatment practices and possible differences in them among CHD patients with and without diabetes.

In this register-based cohort study that included all patients who underwent a coronary revascularisation operation between 1998 and 2007 in Finland, we examined the time trends of coronary revascularisation operations and compared patients with diabetes to non-diabetic CHD patients. Furthermore, we explored whether the increase in revascularisation rates was associated with treatment pathways leading to revascularisation.

## Methods

### Patient population

The study is based on individual-level nationwide register data on CHD patients with or without diabetes who underwent a coronary revascularisation operation between January 1, 1998 and December 31, 2007 in Finland. This study was approved by the ethics committee of the National Institute for Health and Welfare. The data linkages were done by appropriate statistical authorities as required by Finnish data protection legislation. The research group received anonymised data in which individuals were not identifiable.

The total population of patients admitted for first coronary artery bypass graft (CABG) or first percutaneous coronary intervention (PCI) were identified in the Finnish Hospital Discharge Register [10]. The Hospital Discharge Register includes all surgical operations delivered in public and private hospitals in Finland. The status of diabetes and other chronic diseases were additionally determined from two national health insurance registers: 1) a register on entitlement to elevated reimbursements for medicine expenditure due to chronic conditions and 2) a register on reimbursed prescriptions. Diabetes type was determined on the basis of prescription data: persons with continuous insulin usage and no purchases of medication intended to increase pancreatic insulin secretion were considered to have insulin dependent diabetes (IDD) and others to have non-insulin dependent diabetes (NIDD). The algorithm used in the identification of chronic comorbidities is described in detail elsewhere [11].

### Defining treatment pathways for analysis

To examine differences in treatment pathways leading to revascularisation, all hospital admissions having CHD as the main diagnosis during the two years preceding the revascularisation operation were included in the study data, and admissions were classified into three categories (1) elective CHD hospital admission, (2) myocardial infarction admission, or (3) other emergency CHD admission. Since emergency admissions signal poor control of CHD, treatment pathways were categorized on the basis of the number of emergency admissions due to CHD or MI before the revascularisation. Treatment pathways were defined as suboptimal if two or more emergency hospitalisations occurred during the two years preceding revascularisation. Furthermore, we examined revascularisation during the first treatment period, i.e. during the first continuous chain of hospitalisations without discharge home. The analysis of treatment pathways is based on an approach we have developed for studying access to hospital care using register-based data [12].

### Statistical analysis

First, we examined trends in the proportions of revascularisations performed during the first treatment period among three patient groups (IDD, NIDD and no diabetes). Second, we examined trends in suboptimal pathways in these patient groups. Third, we modelled the probability of revascularisation in the first treatment period and suboptimal treatment pathways. In order to control for confounding due to cluster effects -- which are potentially caused by the multilevel structure of the data where patients are nested within regions (municipalities) -- we used conditional logistic regression modelling with region as a stratum [13]. The basic models were also adjusted for age and type of revascularisation. We also estimated models with further adjustment for comorbidities. Odds ratios and their 95% confidence intervals were calculated for different patient groups and time periods. All analyses were performed for men and women separately.

## Results

Distributions of the study variables for the CHD population with the first coronary revascularisation are presented in Table 1. In total we analyzed 78 774 pathways leading to first revascularisations for the period 1998-2007 in Finland, with the majority performed in the age group 55-69. The number of first CABGs was 27 654 among men and 9514 among women. The first PCI was performed for 28 485 men and for 13 121 women. Hypertension was the most common comorbidity. The average number of hospitalisations due to CHD during the two years preceding revascularisation was 1.64 among men and 1.62 among women. The number of CHD hospitalisations was higher among diabetic than non-diabetic patients. During the study period, the average number of CHD hospitalisations in the two years period prior to revascularisation decreased in both genders and all patient groups. The means decreased from 2.04 (CI 95% 2.02-2.06) in 1998-2000 to 1.31 (CI 95% 1.30-1.32) in 2005-2007 among men with CHD without diabetes; from 2.51 (CI 95% 2.31-2.71) in 1998-2000 to 1.41 (CI 95% 1.39-1.43) in 2005-2007 among male patients with IDD; and from 2.23 (CI 95% 2.22-2.25) in 1998-2000 to 1.41 (CI 95% 1.39-1.42) in 2005-2007 among male patients with NIDD. Among women, the means decreased from 1.96 (CI 95% 1.93-1.99) in 1998-2000 to 1.31 (CI 95% 1.30-1.33) in 2005-2007 among those with CHD without diabetes; from 2.11 (CI 95% 1.93-2.29) in 1998-2000 to 1.50 (CI 95% 1.40-1.59) in 2005-2007 among female patients with IDD; and from 2.19 (CI 95% 2.12-2.27) in 1998-2000 to 1.42 (CI 95% 1.38-1.45) in 2005-2007 among female patients with NIDD.

**Table 1:** Background characteristics of CHD population in Finland 1998–2007.

		Men						Women					
		No diabetes (n)	%	IDD (n)	%	NIDD (n)	%	No diabetes (n)	%	IDD (n)	%	NIDD (n)	%
Revascularisations year(s):	1998-2007	44 498		1 121		10 520		16 731		651		5 253	
	1998	3 818		92		678		1 247		46		377	
	2007	4 422		152		1 202		1 807		74		625	
Revascularisations type:	PCI	23 290	52	506	45	4 689	45	9 925	59	343	53	2 853	54
	CABG	21 208	48	615	55	5 831	55	6 806	41	308	47	2 400	46
Age:	-39	728	2	54	5	59	1	170	1	42	6	26	0
	40-54	9 461	21	375	33	1 396	13	1 516	9	204	31	277	5
	55-69	21 093	47	501	45	5 444	52	6 229	37	249	38	1 848	35
	70+	13 216	30	191	17	3 621	34	8 816	53	156	24	3 102	59
Comorbidities:													
	Hypertension	23 495	53	881	79	8 102	77	11 287	67	536	82	4 484	85
	Atrial fibrillation	3 261	7	45	4	1 046	10	1 217	7	29	4	536	10
	Cardiac failure	2 730	6	145	13	1 266	12	1 421	8	94	14	964	18
	Alcoholism/drug abuse	963	2	39	3	214	2	87	1	11	2	26	0
	Cancer	2 554	6	54	5	663	6	1 100	7	27	4	382	7
	COPD and asthma	5 104	11	135	12	1 436	14	2 611	16	97	15	958	18
	Depression	611	1	23	2	176	2	263	2	15	2	119	2
	Parkinson's disease	274	1	9	1	98	1	147	1	8	1	66	1
	Mental disorder	1 031	2	35	3	364	3	492	3	29	4	255	5
	Dementia	105	0	3	0	37	0	67	0	2	0	22	0
	Renal insufficiency	119	0	76	7	50	0	29	0	33	5	22	0
Number of CHD hospitalizations, mean (standard deviation)		1.62	(0.86)	1.87	(1.15)	1.72	(0.99)	1.59	(0.87)	1.71	(0.92)	1.70	(1.04)

Abbreviations: No diabetes, patients without diabetes; IDD, insulin dependent diabetes; NIDD, non-insulin dependent diabetes; PCI, percutaneous coronary intervention; CABG, coronary artery bypass graft; Number of CHD hospitalizations covers two years period preceding revascularization

The distributions of CHD hospital admissions in terms of urgency were similar among patients with and without diabetes: Among men with diabetes, 50% of the hospital admissions that were due to CHD were elective, 27% were myocardial infarction admissions, and 23% were other emergency admissions for CHD. Among men without diabetes the proportions were 51%, 25% and 24%, respectively. Among women with diabetes 42% were elective CHD hospital admissions, 28% myocardial infarction admissions, and 30% were other emergency CHD admissions, while for women without diabetes the proportions were 46%, 26% and 28%, respectively.

**Figure 1:**
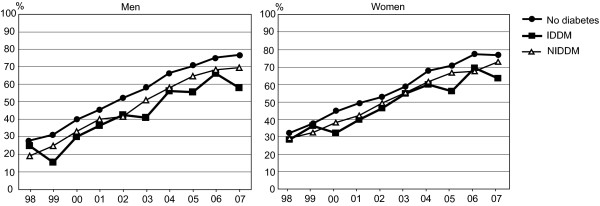
**Trends in coronary revascularisation performed at the first treatment period in 1998-2007**.

The proportion of coronary revascularisations performed during the first treatment period increased systematically from 1998 to 2007 among both men and women, while the proportions were on a rather similar level among both genders (Figure 1). The proportions of revascularisation operations in the first treatment period were smaller among patients with diabetes compared to those without (p < 0.001) revascularisation

**Figure 2:**
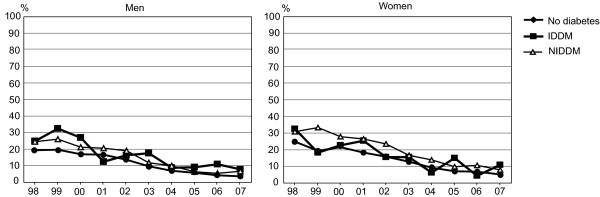
**Trends in suboptimal pathways leading to coronary revascularisation in 1998-2007**.

While revascularisations in the first treatment period increased, the number of suboptimal treatment spells decreased by at least two-thirds in all patient groups. A clear downward trend of the proportions of suboptimal treatment pathways were found both among men and women (Figure 2), but among patients with diabetes, suboptimal treatment spells were more probable (p < 0.001).

Among men the odds for revascularisation in the first hospital treatment period were lower among patients with diabetes compared to those without diabetes in the period 1998-2000 (Table 2), with the results remaining the same even after further adjustment for comorbidity. Similar differences in the odds for revascularisation in the first hospital admission were found between women with and without diabetes in 1998-2000, but the differences did not reach statistical significance. During the study period the proportion of revascularisations in the first hospital admission increased rapidly in both genders and in all patient groups. In 2005-2007 among men and women the odds for revascularisation in the first hospital treatment period were lower among patients with diabetes than patients without diabetes. The adjustment for comorbidities did diminish the differences, but it did not remove them entirely.

**Table 2:** Odds-ratios for revascularisation at first CHD hospitalisation by gender and history of diabetes.

		Men				Women			
		Age adjusted		Comorbidity adjusted		Age adjusted		Comorbidity adjusted	
		OR	95% CI	OR	95% CI	OR	95% CI	OR	95% CI
1998-2000	No diabetes	1.00	ref.	1.00	ref.	1.00	ref.	1.00	ref.
	IDD	0.66	(0.49-0.89)	0.73	(0.54-0.99)	0.68	(0.47-1.00)	0.76	(0.52-1.11)
	NIDD	0.80	(0.71-0.89)	0.86	(0.77-0.96)	0.88	(0.77-1.02)	0.95	(0.83-1.10)
2005-2007	No diabetes	1.00	ref.	1.00	ref.	1.00	ref.	1.00	ref.
	IDD	0.59	(0.48-0.73)	0.52	(0.42-0.64)	0.53	(0.40-0.70)	0.59	(0.44-0.78)
	NIDD	0.86	(0.80-0.94)	0.79	(0.73-0.86)	0.77	(0.69-0.87)	0.83	(0.74-0.93)

All models are adjusted for age, type of revascularization (PCI or CABG), and region. Additional comorbidity adjustment is done for hypertension, atrial fibrillation, cardiac failure, alcoholism or drug abuse, cancer, COPD and asthma, depression, Parkinson's disease, psychotic mental disorders, dementia, and renal insufficiency. Abbreviations: No diabetes, patients without diabetes; IDD, insulin dependent diabetes; NIDD, non-insulin dependent diabetes; OR, odds ratio; CI, confidence interval

In 1998-2000, men with diabetes were at a higher risk for suboptimal pathways to revascularisation than those without diabetes (Table 3). The difference was especially large among patients with IDD. Even though the adjustment for comorbidity decreased these differences in both groups of patients with diabetes it did not abolish them entirely. In 2005-2007, men with diabetes still had a higher risk for suboptimal pathways to revascularisation than CHD patients without diabetes. Although controlling for comorbidities diminished the differences, they remained statistically significant among patients with IDD. In 1998-2000, women with diabetes were more at risk for suboptimal pathways to revascularisation, even after adjusting for comorbidity. The differences remained even in the period 2005-2007.

**Table 3:** Odds-ratios for suboptimal pathway leading to revascularisation by gender and history of diabetes.

		Men				Women			
		Age adjusted		Comorbidity adjusted		Age adjusted		Comorbidity adjusted	
		OR	95% CI	OR	95% CI	OR	95% CI	OR	95% CI
1998-2000	No diabetes	1.00	ref.	1.00	ref.	1.00	ref.	1.00	ref.
	IDD	2.02	(1.52-2.67)	1.67	(1.26-2.22)	1.79	(1.18-2.70)	1.52	(1.00-2.30)
	NIDD	1.29	(1.15-1.44)	1.12	(1.00-1.26)	1.44	(1.24-1.67)	1.28	(1.10-1.49)
2005-2007	No diabetes	1.00	ref.	1.00	ref.	1.00	ref.	1.00	ref.
	IDD	2.46	(1.74-3.46)	1.95	(1.38-2.76)	2.29	(1.46-3.57)	1.91	(1.22-3.00)
	NIDD	1.28	(1.10-1.49)	1.12	(0.96-1.30)	1.47	(1.22-1.77)	1.30	(1.08-1.57)

All models are adjusted for age and type of revascularization (PCI or CABG), and region. Additional comorbidity adjustment is done for hypertension, atrial fibrillation, cardiac failure, alcoholism or drug abuse, cancer, COPD and asthma, depression, Parkinson's disease, psychotic mental disorders, dementia, and renal insufficiency. Abbreviations: No diabetes, patients without diabetes; IDD, insulin dependent diabetes; NIDD, non-insulin dependent diabetes; OR, odds ratio; CI, confidence interval

## Discussion

This study was based on nationwide register data that included all CHD patients who underwent coronary revascularisation. It examined hospital treatment pathways leading to the operation in Finland between 1998 and 2007. More specifically, we examined the treatment pathways among CHD patients with diabetes during a period that saw a large increase in revascularisation operations. Overall, treatment practices changed substantially during the study period to favour performing revascularisation operations during the first hospital admission, whether it was an emergency admission or not. This was true for both men and women and for patients with and without diabetes. The large increase in PCIs is likely to be an important factor behind these changes, as it is likely that CABG will be more often planned for a subsequent hospitalisation than PCI.

However, patients with diabetes admitted to coronary revascularisation received relatively fewer operations in the first CHD hospital admission compared to their counterparts without diabetes. The differences remained rather stable during the whole study period. While suboptimal treatment pathways decreased substantially during the study period, pathways including two or more emergency admissions were still more common among patients with diabetes at the end of the study period. One explanation might be that health services provide a lower threshold for CHD admission among patients with diabetes. The average number of hospitalisations due to CHD was higher among patients with diabetes. However, distributions of categories for CHD hospitalisations were similar in both patient groups. Of all CHD admissions of men and women with and without diabetes, a quarter were due to MI, which was obviously diagnosed by the same criteria for all patients, that is using a blood test (troponin-T) with high sensitivity and specificity. In Finnish hospitals this test was introduced in 1997-2000 [14]. Other emergency CHD admissions comprised a quarter of men's and a third of women's admissions among both diabetic and non-diabetic patients. Therefore we consider that it is unlikely that health services provide a substantially different threshold for CHD admissions for diabetic patients compared to non-diabetic patients. Neither do these differences in suboptimal treatment pathways seem to be solely due to a different case-mix among patients with and without diabetes, since taking into account differential comorbidity between the patient groups did not eliminate the differences. We also adjusted for the type of revascularisation in our models, and that did not explain the differences. Adjustment for region using conditional logistic regression assured that confounding due to cluster effects was adequately controlled.

We made further sensitivity analyses by adjusting for the MI during the two years period preceding the operation. These analyses revealed that persons with MI had clearly higher odds to undergo revascularisation at their first hospitalisation, and slightly higher odds for the suboptimal pathways leading to CABG. The differences between patients with and without diabetes, however, remained the same or slightly increased after adjustment for MI. The interpretations of the results did not change, but these sensitivity analyses suggest that our findings were rather conservative.

Adjusting for changes in the characteristics of the patient population over time, we modelled specific time periods at the beginning and end of study, comparing at both time points the non-diabetic patients to those with IDD or NIDD. Possible changes in the patient population included an increase in the number of patients with diabetes [15], a shift in CHD incidence towards elderly persons, an increased number of revascularisations, and a more precise technology used in determining the need for revascularisation.

The ten-year study period allowed us to examine changes in treatment practices among both diabetic and non-diabetic patient groups. Since the data cover all public and private hospitals, we were able to examine the total population of patients undergoing a coronary revascularisation operation during the study period.

The quality and coverage of the Finnish Hospital Discharge Register has been reported to be generally good and particularly good among patients with MI [16,17]. Information on chronic diseases was also obtained from two other registers: a register on the reimbursement of prescriptive medicine costs and a register on persons eligible for elevated mandatory health insurance reimbursement of drug costs. Since the prescription register is an administrative register based on actual reimbursements of medicine costs, its coverage is likely to be very high. Eligibility for an elevated level of reimbursement requires a doctor's certificate confirming that the criteria set by the Social Insurance Institution are met, with the certificate reviewed by a medical specialist at the Social Insurance Institution. The use of all three registers allowed for a reliable identification of chronic diseases.

When interpreting the results, it is important to bear in mind that the study population is a retrospective cohort of hospitalized CHD patients who underwent a coronary revascularisation operation. Our study cannot therefore estimate whether access to revascularisation is inequitable among persons with diabetes compared with other coronary patients. An earlier study from Finland does suggest that diabetes decreases the likelihood of coronary revascularisation [18]. Additionally, the diabetes population has clearly higher mortality compared to others [19,20]. Based on our data, we cannot estimate the proportion of those in need of revascularisation, who died before the operation, or who were not revascularised in spite of a need. The issue of equitable access to revascularisation among patients with and without diabetes is complex, and there is an ongoing discussion on whether an initial strategy of revascularisation, or a conservative approach with drugs is most effective for coronary patients with diabetes [21].

Earlier studies have mainly examined differences in access to revascularisation instead of pathways to it and to our knowledge similar studies are scarce. A cohort study of MI patients admitted to Californian hospitals found both ethnic and payer group differences in treatment pathways; uninsured and minority patients were less likely to have treatment pathways leading to revascularisation. They were less likely to receive the operation at the first admission, to be transferred to a hospital offering revascularisation or to be readmitted to undergo revascularisation [22]. Other retrospective studies of selection to health care in CHD (revascularisation and cardiac rehabilitation) suggest there are barriers in treatment pathways among older patients, women and lower socioeconomic groups [23-25].

Our retrospective study design enabled us to examine treatment pathways leading to revascularisation in different patient groups in terms of hospital care. Earlier research has reported socioeconomic differences in access to revascularisation among coronary patients in general as well as patients with diabetes [7,18,26,27]. Further research is needed on the potential socioeconomic differences in pathways to operations among patients with diabetes.

Differences between patient groups in the revascularisations performed in the first treatment period may partly echo delays in clinical decision-making on referral for revascularisation. Due to complicating conditions requiring medical attention, diabetic CHD patients and especially patients with IDD may experience these delays more often than CHD patients without diabetes. The registers used in this study lack information on these clinical details. However, allowing for delays in decision-making, suboptimal treatment pathways should not be more common among patients with diabetes. Moreover, the differences in operations at the first CHD admission as well as in suboptimal pathways remained even after adjusting for observable comorbidities. In conclusion, our results suggest that the differences in the risk of suboptimal treatment pathways may partly be explained by treatment practices that are not in accordance with the evidence-based treatment guidelines that suggest that the benefits of revascularisation are similar in both patients groups.

## Conclusions

In general, the number of coronary revascularisations rose in parallel with increased resources and with changed treatment practices, putting more emphasis on performing revascularisations on CHD patients during the first hospital admission. However, among men with IDD and women with IDD and NIDD, the excess risk for suboptimal pathways to coronary revascularisation remained. These results are in line with previous studies reporting suboptimal use of lipid-lowering medication to prevent CHD among patients with diabetes [6]; furthermore, while CHD mortality has declined constantly, the excess mortality among patients with diabetes compared to the general population has not decreased in recent years [28]. More attention should be focused on cardiac care among patients with diabetes in terms of secondary prevention of adverse cardiac events.

## Abbreviations

CABG: coronary artery bypass graft; CHD: coronary heart disease; CI: confidence interval, IDD insulin dependent diabetes; MI: myocardial infarction; NIDD: non-insulin dependent diabetes; OR: odds ratio; PCI: percutaneous coronary intervention

## Competing interests

The authors declare that they have no competing interests.

## Authors' contributions

TV wrote the manuscript and analyzed data, RS made the statistic analyses and wrote and reviewed the manuscript, KM reviewed the manuscript and contributed to the discussion, UH researched data and reviewed the manuscript, IK reviewed the manuscript and contributed to the discussion. All authors read and approved the final manuscript.

## Pre-publication history

The pre-publication history for this paper can be accessed here:

http://www.biomedcentral.com/1472-6963/11/180/prepub

## References

[B1] Alter DA Khaykin Y Austin PC Tu JV Hux JE Processes and outcomes of care for diabetic acute myocardial infarction patients in Ontario: do physicians undertreat? Diabetes Care 2003 26 5 1427 1434 10.2337/diacare.26.5.1427 12716800

[B2] Norhammar A Malmberg K Ryden L Tornvall P Stenestrand U Wallentin L Register of Information and Knowledge about Swedish Heart Intensive Care Admission (RIKS-HIA) Under utilisation of evidence-based treatment partially explains for the unfavourable prognosis in diabetic patients with acute myocardial infarction Eur Heart J 2003 24 9 838 844 10.1016/S0195-668X(02)00828-X 12727151

[B3] Gitt AK Schiele R Wienbergen H Zeymer U Schneider S Gottwik MG Senges J for the MITRA Study Group Intensive treatment of coronary artery disease in diabetic patients in clinical practice: results of the MITRA study Acta Diabetol 2003 40 S343 S347 10.1007/s00592-003-0117-8 14704866

[B4] Salomaa V Pääkkönen R Hämäläinen H Niemi M Klaukka T Use of secondary preventive medications after the first attack of acute coronary syndrome Eur J Cardiovasc Prev Rehabil 2007 14 386 391 10.1097/01.hjr.0000244573.10229.6e 17568237

[B5] Manderbacka K Keskimäki I Reunanen A Klaukka T Equity in the use of antithrombotic drugs, beta-blockers and statins among Finnish coronary patients Int J Equity Health 2008 30 7 16 10.1186/1475-9276-7-16 PMC2459171 18590524

[B6] Vehko T Manderbacka K Arffman M Sund R Reunanen A Keskimäki I Changing patterns of secondary preventive medication among newly diagnosed coronary heart disease patients with diabetes in Finland: a register-based study Scand J Public Health 2010 38 3 317 324 10.1177/1403494810364558 20228159

[B7] Hetemaa T Keskimäki I Salomaa V Mähönen M Manderbacka K Koskinen S Socioeconomic inequities in invasive cardiac procedures after first myocardial infarction in Finland in 1995 J Clin Epidemiol 2004 57 3 301 8 10.1016/j.jclinepi.2003.07.010 15066691

[B8] Saaristo T Peltonen M Keinänen-Kiukaanniemi S Vanhala M Saltevo J Niskanen L Oksa H Korpi-Hyövälti E Tuomilehto J for the FIN-D2D Study Group National Type 2 Diabetes Prevention Programe in Finland: FIN-D2D Int J Circumpolar Health 2007 66 2 101 112 10.3402/ijch.v66i2.18239 17515250

[B9] Ryden L Standl E Bartnik M Van den Berghe G Betteridge J de Boer MJ Cosentino F Jonsson B Laakso M Malmberg K Priori S Ostergren J Tuomilehto J Thrainsdottir I Vanhorebeek I Stramba-Badiale M Lindgren P Qiao Q Priori SG Blanc JJ Budaj A Camm J Dean V Deckers J Dickstein K Lekakis J McGregor K Metra M Morais J Osterspey A Tamargo J Zamorano JL Deckers JW Bertrand M Charbonnel B Erdmann E Ferrannini E Flyvbjerg A Gohlke H Juanatey JR Graham I Monteiro PF Parhofer K Pyörälä K Raz I Schernthaner G Volpe M Wood D Task Force on Diabetes and Cardiovascular Diseases of the European Society of Cardiology (ESC) European Association for the Study of Diabetes (EASD) Guidelines on diabetes, pre-diabetes, and cardiovascular diseases: executive summary. The Task Force on Diabetes and Cardiovascular Diseases of the European Society of Cardiology (ESC) and of the European Association for the Study of Diabetes (EASD) Eur Heart J 2007 28 1 88 136 17220161 10.1093/eurheartj/ehl260

[B10] Seppälä T Hartikainen J Häkkinen U Juntunen M Linna M Nikus K Pelanteri S Peltola M Rauhala A Vento A PERFECT - Pallolaajennus ja ohitusleikkaus: Toimenpiteiden kustannukset ja vaikuttavuus tuottajatasolla [In Finnish: PERFECT (Performance, Effectiveness and Cost of Treatment episodes -project) - Coronary revascularizations, cost and effectiveness] 2008 Helsinki: National Research and Development Centre for Welfare and Health

[B11] Juntunen M Sund R Peltola M Häkkinen U Potilasrakenteiden erojen huomioon ottaminen erikoissairaanhoidon vaikuttavuuden rekisteritutkimuksessa (In Finnish with an English abstract: Risk-adjustment in register -based evaluation of hospital care) Sosiaalilääketieteellinen Aikakauslehti [Journal of Social Medicine in Finland] 2008 4 258 272

[B12] Sund R Kajantie M Manderbacka K Keskimäki I Socioeconomic inequities in treatment pathways leading to coronary revascularization in Finland in 1995-1998 EJPH 2007 17 Supplement 2 128

[B13] Berlin JA Kimmel SE Ten Have TR Sammel MD An empirical comparison of several clustered data approaches under confounding due to cluster effects in the analysis of complications of coronary angioplasty Biometrics 1999 55 2 470 476 10.1111/j.0006-341X.1999.00470.x 11318202

[B14] Salomaa V Koukkunen H Ketonen M Immonen-Räiha P Karja-Koskenkari P Mustonen J Lehto S Torppa J Lehtonen A Tuomilehto J Kesäniemi YA Pyörälä K A new definition for myocardial infarction: what difference does it make? Eur Heart J 2005 26 17 1719 1725 10.1093/eurheartj/ehi185 15814567

[B15] Reunanen A Koskinen S, Aromaa A, Huttunen J, Teperi J Diabetes Health in Finland 2006 National Public Health Institute, Stakes, Ministry of Social Affairs and Health 176

[B16] Gissler M Haukka J Finnish health and social welfare registers in epidemiological research Norsk Epidemiologi 2004 14 1 113 120

[B17] Pajunen P Koukkunen H Ketonen M Jerkkola T Immonen-Räihä P Karja-Koskenkari P Mähönen M Niemelä M Kuulasmaa K Palomäki P Mustonen J Lehtonen A Arstila M Vuorenmaa T Lehto S Miettinen H Torppa J Tuomilehto J Kesäniemi YA Pyörälä K Salomaa V The validity of the Finnish Hospital Discharge Register and Causes of Death Register data on coronary heart disease Eur J Cardiovasc Prev Rehabil 2005 12 2 132 7 15785298 10.1097/00149831-200504000-00007

[B18] Hetemaa T Manderbacka K Reunanen A Koskinen S Keskimäki I Socioeconomic inequities in invasive cardiac procedures among patients with incident angina pectoris or myocardial infarction Scand J Public Health 2006 34 2 116 23 10.1080/14034940510032248 16581703

[B19] Forssas E Reunanen A Keskimäki I Koskinen S Coronary heart disease among diabetic and nondiabetic people -- socioeconomic differences in incidence, prognosis and mortality J Diabetes Complications 2008 22 1 10 17 10.1016/j.jdiacomp.2007.05.004 18191072

[B20] Haffner SM Lehto S Rönnemaa T Pyörälä K Laakso M Mortality from coronary heart disease in subjects with type 2 diabetes and nondiabetic subjects with and without prior myocardial infarction N Engl J Med 1998 339 4 229 234 10.1056/NEJM199807233390404 9673301

[B21] Soares PR Hueb WA Lemos PA Lopes N Martinez EE Cesar LA Oliveira SA Ramires JA Coronary revascularization (surgical or percutaneous) decreases mortality after the first year in diabetic subjects but not in nondiabetic subjects with multivessel disease: an analysis from the Medicine, Angioplasty, or Surgery Study (MASS II) Circulation 2006 114 1 Suppl I420 4 16820611 10.1161/CIRCULATIONAHA.105.000679

[B22] Blustein J Arons RR Shea S Sequential events contributing to variations in cardiac revascularization rates Med Care 1995 33 8 864 880 10.1097/00005650-199508000-00010 7637407

[B23] Bowling A Bond M McKee D McClay M Banning AP Dudley N Elder A Martin A Blackman I Equity in access to exercise tolerance testing, coronary angiography, and coronary artery bypass grafting by age, sex and clinical indications Heart 2001 85 6 680 686 10.1136/heart.85.6.680 11359752 PMC1729768

[B24] Williams JAS Byles JE Inder KJ Equity of access to cardiac rehabilitation: the role of system factors Int J Equity Health 2010 9 2 10.1186/1475-9276-9-2 PMC2823593 20205776

[B25] Alter DA Iron K Austin P Naylor CD for the SESAMI Study Group Socioeconomic status, service patterns, and perceptions of care among survivors of acute myocardial infarction in Canada JAMA 2004 291 9 1100 1107 10.1001/jama.291.9.1100 14996779

[B26] Hetemaa T Keskimäki I Manderbacka K Leyland A Koskinen S How did the recent increase in the supply of coronary operations in Finland affect socioeconomic and gender equity in their use? J Epidemiol Community Health 2003 57 3 178 85 10.1136/jech.57.3.178 12594194 PMC1732404

[B27] Vehko T Manderbacka K Arffman M Reunanen A Keskimäki I Increasing resources effected equity in access to revascularizations for patients with diabetes Scand Cardiovasc J 2010 44 4 237 244 10.3109/14017431.2010.494309 20586656

[B28] Forssas E Sund R Manderbacka K Arffman M Ilanne-Parikka P Keskimäki I Diabeetikoilla yhä suuri ylikuolleisuus muuhun väestöön verrattuna (In Finnish with an English abstract: Excess mortality has remained high among people with diabetes) Suom Laakaril 2010 26 31 2359 2367

